# Axodendritic targeting of TAU and MAP2 and microtubule polarization in iPSC-derived versus SH-SY5Y-derived human neurons

**DOI:** 10.1515/biol-2022-1010

**Published:** 2024-12-31

**Authors:** Helen Breuer, Michael Bell-Simons, Hans Zempel

**Affiliations:** Institute of Human Genetics, Faculty of Medicine and University Hospital Cologne, University of Cologne, Kerpener Str. 34, 50931, Cologne, Germany; Center for Molecular Medicine Cologne (CMMC), University of Cologne, Robert-Koch-Str. 21, 50931, Cologne, Germany; Current address: Max-Planck-Institute for Aging, Joseph-Stelzmann-Straße 11, 50931, Cologne, Germany; Department of Nuclear Medicine, Faculty of Medicine and University Hospital Cologne, University of Cologne, 50937, Cologne, Germany

**Keywords:** neuronal cell polarity, EB3-trafficking, dendrite, axon-initial-segment, AnkG/TRIM46

## Abstract

Cell polarity is crucial in neurons, characterized by distinct axonal and dendritic structures. Neurons generally have one long axon and multiple shorter dendrites, marked by specific microtubule (MT)-associated proteins, e.g., MAP2 for dendrites and TAU for axons, while the scaffolding proteins AnkG and TRIM46 mark the axon-initial-segment. In tauopathies, such as Alzheimer’s disease (AD), TAU sorting, and neuronal polarity are disrupted, leading to MT loss. However, modeling and studying MTs in human neuronal cells relevant to the study of AD and TAU-related neurodegenerative diseases (NDD) is challenging. To study MT dynamics in human neurons, we compared two cell culture systems: SH-SY5Y-derived neurons (SHN) and induced pluripotent stem cell-derived neurons (iN). Using immunostaining and EB3-tdTomato time-lapse imaging, we found AnkG absent in SHN but present in iN, while TRIM46 was present in both. TAU and MAP2 showed axonal and dendritic enrichment, respectively, similar to mouse primary neurons. Both neuron types exhibited polarized MT structures, with unidirectional MTs in axons and bidirectional MTs in dendrites. Polymerization speeds were similar; however, iNs had more retrograde MT growth events, while SHN showed a higher overall number of growth events. Thus, SHN and iN are both suitable for studying neuronal cell polarity, with SHN being particularly suitable if the focus is *not* the AIS.

## 1 Introduction

TAU is a major microtubule-associated protein (MAP) in the human brain, where it contributes to microtubule (MT) stabilization and regulates MT dynamics. Normally, TAU is sorted and restricted mainly to the axon, where it is associated with MTs. In pathological conditions, it can dissociate from MTs, mislocalize into the somatodendritic compartment, and form insoluble aggregates, known as neurofibrillary tangles (NFTs). These NFTs are the hallmark lesions of tauopathies, which include Alzheimer’s disease (AD), frontotemporal dementia, atypical Parkinson’s syndromes like corticobasal degeneration, progressive supranuclear palsy, and other tauopathies [1,2]. Missorted TAU leads to or coincides with the breakdown of the MT system, compromised axonal transport, synaptic dysfunction, and neurodegeneration [3,4,5]. Much is known about the downstream effects of missorted TAU, but the upstream events that lead to TAU missorting or the mechanisms enabling physiological sorting under healthy conditions are poorly understood.

In the case of AD, we know that the loss of TAU’s MT-stabilizing function and its downstream effects constitute a distinct cause leading to neurodegeneration, aside from the toxic gain-of-function of TAU aggregates [6]. Besides AD, many other neurodegenerative diseases display the effects of a disrupted MT network – for example, intracellular protein aggregates disturb axonal transport in synucleinopathies, ALS or Huntington’s disease [7], and defects of motor proteins lead to intellectual disabilities [8]. Moreover, mutations in MT-related genes cause defective cell migration, as seen in lissencephaly, or hinder MT-cytoskeletal rearrangements during neuronal development, like in autism spectrum disorders [8,9,10].

Importantly, MTs in neurons have a particular organization that differs significantly from other cell types and also within the compartments of the neuron itself [11,12]. The minus end is anchored in the MTOC, which is classically the centrosome in most dividing eukaryotic cells. In spherical somatic cells, e.g., fibroblasts or unpolarized neuronal progenitors, MTs would classically grow out centrifugally from a central centrosome. In postmitotic neurons, the centrosome loses its function as an MTOC during development, and instead, the MTs grow out from Golgi outposts in dendrites or by non-centrosomal MTOC proteins, e.g., on behalf of augmin from pre-existing MTs or from the MAP CAMSAP2 at the proximal axon [13,14].

Aside from atypical growth origins, MTs have particular features differing between axons and dendrites, including their orientation, stability, bundling, and chemical tubulin modifications [12]. MTs in the axon are uniformly orientated with their plus end outwards, thus growing anterogradely from the soma, while MTs in the dendrite show a mixed polarity, with a varying fraction of anterograde and retrograde growing MTs depending on the species and neuronal cell type [15]. In the dendrite, anterograde MTs make up a portion of approximately 65% in mice *in vivo* [16], 11% in *Drosophila* [17], 55% in hippocampal rat neurons [18], and 85% in human [19]. The different orientation in axons and dendrites seems to be important for two mechanisms: (a) the uniform orientation of MTs in the axon is important for the axonal outgrowth and specification [18] and (b) the different polarity of MTs directly drives polarized cargo transport. Kinesin-driven cargo is directed towards the plus end and targeted primarily to the axon, whereas dynein-driven vesicles drive toward the minus end, preferentially on dendritic MTs [20,21]. This is important because the cargos themselves contain proteins that determine the axonal or dendritic fate, e.g., synaptic receptors for the axonal synapse.

Neuronal MT dynamics are being studied i.a. in primary neurons derived from rodents, the standard model for mammalian neurobiology *in vitro*. Unfortunately, human disease is only partially recapitulated in rodents and rodent-based human disease models, and there are notable differences in gene and protein sequences and isoform composition of disease-associated players, which is in particular true for TAU [22]. This makes it difficult to draw conclusions from rodent models, but primary human neurons are very rarely available (e.g., from abort material or from neurosurgically removed peripheral ganglia in case of injury) and unsuitable for research purposes. With biotechnological progress over the past two decades, inducible pluripotent stem cells (iPSCs) can now be differentiated into various types of human cells, including neurons and their numerous subtypes [23,24,25]. Somatic cells are genetically reprogrammed into pluripotent iPSCs, which can be transformed into neuronal precursor cells and finally neurons, by exposing them to external morphogens – a method called “directed differentiation” – or via genetic fating usually achieved by induced expression of a neurotransmitter driving differentiation into specific neuronal subtypes [26,27].

An alternative human neuronal cell system, besides induced pluripotent stem cell (iPSC)-derived neurons (iN), are secondary neuronal cell lines derived from neuronal tumors. An extensively used type is the human neuroblastoma cell line SH-SY5Y. Treatment of this cell line with several chemicals, including retinoic acid (RA), brain-derived neurotrophic factor (BDNF), neuronal growth factor, or others, results in the generation of excitatory neuronal cells [28,29,30,31]. SH-SY5Y cells can be differentiated within 2–3 weeks in cultures showing neuronal morphology and neuronal markers [30,32,33]. Undifferentiated SH-SY5Y neuroblastoma cells show catecholaminergic characteristics but during differentiation, they develop into neurons inheriting cholinergic, noradrenergic, and dopaminergic properties, of which all three neuronal subtypes are present in AD-affected loci [29,30]. As pre-mitotic cells, SH-SY5Y cells undergo cell division and can provide a vast quantity of neuronal cells for experimental arrays. Their differentiation is less time- and cost-consuming than those of iN and yields cultures of higher homogeneity and robustness.

Here, we set out to test MT dynamics in iN and SH-SY5Y-derived neurons (SHN) and in different neuronal subcompartments. We first tested proper neuronal compartmentalization, i.e., axodendritic development, axon-initial-segment (AIS) establishment, and polarized sorting of TAU and MAP2. We compared the efficiency of axonal sorting of TAU and MAP2, and expression of the AIS-marker proteins AnkG and TRIM46 in iN to SHN and the standard model of neurobiology, mouse primary neurons (mPN). We found that iN and SHN showed remarkably similar TAU sorting properties, which were also comparable to mPN, but SHN lacked expression of AnkG and showed reduced expression of TRIM46. We then investigated MT dynamics via EB3-live-imaging and found that i.a. MTs are orientated anterogradely in axons of both iN and SHN, but more retrogradely in iN dendrites. MT polymerization speed was much alike in the three subcompartments of SHN and iN, whereas in SHN, we observed generally more growth events. Conclusively, overall cell polarization and neuronal compartmentalization do take place in SHN and iN, but only iN express and enrich AnkG and TRIM46 at the AIS and their MT-dynamics appear more reminiscent of mature neurons.

## 2 Methods

### 2.1 Cell culture

#### 2.1.1 Cultivation

Both SH-SY5Y cells and iPSCs followed a differentiation protocol into neuronal cell cultures with a duration of 2–3 weeks. Both cell models were cultivated in 6-well, 12-, and 24-well plates (VWR) or in imaging dishes (ibidi). Well plates have partly been equipped with coverslips (VWR) for microscopy experiments. If not stated otherwise, the amount of solutions and media was 2 ml for a well of a six-well plate, 1 ml for a well of a 12-well plate and 0.5 ml for a well of a 24-well plate. Media reagents were prewarmed at 37°C and cells were kept in a humidified incubator at 37°C with 5% CO_2_.

### 2.2 SY-SY5Y cultivation

Undifferentiated SH-SY5Y cells were cultivated in DMEM/F-12 GlutaMAX™ (# 10565-018, TFS) supplemented with 10% fetal bovine serum (FBS, Biochrom AG), and Antibiotic–Antimycotic (1X Anti/Anti, # 15240062, TFS) (referred to as SHM-10 medium) in uncoated T75 flasks (VWR). SHM-10 medium was changed twice per week and SH-SY5Y cells were passaged when reaching 80% confluence. For passaging cells were washed with phosphate-buffered saline (PBS, TFS), trypsinized (0.05% trypsin/EDTA, TFS) for 3–5 min, and centrifuged for 3 min at 1,000 × *g*. The cell pellet was re-suspended in 10 ml SHM-10 medium, diluted to 1:10, and seeded for further cultivation.

For differentiation, SH-SY5Y was seeded onto plates or imaging dishes, previously coated with 20 µg/ml poly-d-lysine in PBS. The PDL coating solution was added to the well plates and incubated for at least 3 h at 37°C. The coating solution was then discarded and wells were washed three times with Dulbecco’s phosphate-buffered saline (DPBS). For imaging experiments, glass coverslips (VWR) were placed into the wells (25 mm for a 6-well plate, 12 mm for a 24-well plate) before adding the coating solution. Seeding density was 8,000 or 5,500 cells/cm^2^ when seeding one, respectively, 2 days prior to the start of differentiation.

### 2.3 SH-SY5Y differentiation

SH-SY5Y cells were differentiated into SHN within 2 weeks performing sequential medium exchanges with the supplements RA and BDNF. SHM-10 medium was exchanged to SHM-10 with 10 μM of RA on days 0, 3, and 5. On day 7 of differentiation, SHM-10 + RA (Sigma-Aldrich) medium was removed, cells were washed once with DPBS and SHM-10 medium without FBS (referred to as “SHM-0”), supplemented with 10 ng/ml BDNF (Peprotech) was added. On day 10, another medium exchange with SHM-0 + BDNF was carried out. On day 14, cell medium was again changed to SHM-10 without supplements. Differentiation was finished at this point with a differentiation efficiency of about 75% and SHN could be used for further experiments. Cultivation continued in SHM-10 medium with medium exchange every 3 days.

### 2.4 Human iPSC (hiPSC)-derived neurons

hiPSCs were transformed into cultures of cortical glutamatergic neurons following a differentiation protocol, adapted from [26]. The used iPSC cell line WTC11 carries a Neurogenin 2 (Ngn2) transgene, inducible by doxycycline, a transcription factor that rapidly converts iPSCs into neurons (for detailed protocol including troubleshooting guide, see [34]).

#### 2.4.1 iPSC cultivation

For routine cultivation, undifferentiated iPSC colonies were grown in cBrew with medium exchanges every 2–3 days on Geltrex-coated (TFS) 6-well plates. Plates were coated on the day of use with 200 μg/ml Geltrex for 30 min at 37°C. Habitually, certain iPSCs differentiated spontaneously before starting induced differentiation and were hence removed manually with a P200 pipette under a sterile hood. iPSCs were passaged when they reached a confluence of about 80%. For passaging, iPSCs were washed with PBS and dissociated with Versene (TFS) for 3–5 min. Versene was aspirated and 1 ml of cBrew was added. Then, the colonies were carefully detached with a cell scraper and the cell suspension was transferred to a 15 ml tube (Falcon). Cell clumps within the suspension were dissolved by trituration with a 5 ml Pasteur pipette. The cell suspension was diluted in cBrew to a routine passaging ratio of about 1:10. Additionally, Thiazovivin (Axon Med Chem) was added in a concentration of 1:5,000 to the cell suspension, which was then distributed to the culture wells. On the next day, the medium was changed to fresh cBrew without Thiazovivin. Cells would typically reach 80% confluency again after 3–4 days.

#### 2.4.2 Differentiation of iPSCs into iN

iPSCs were transformed into cortical glutamatergic neuronal cultures following a 3-day pre-differentiation (d3 to d0), followed by a multiple-week-long differentiation (d0 to d21 or further) protocol. Three days before starting differentiation (d3), iPSCs were passaged onto Geltrex-coated plates. Cells were washed once with DPBS and then dissociated with Accutase (Merck). Cells were incubated in Accutase for 5–8 min at 37°C. Dissociation was stopped by adding 3 ml of DPBS, then cells were collected in a 15 ml tube (Falcon) and centrifuged for 5 min at 400 × *g*. The supernatant was discarded and the cell pellet was re-suspended in 1 ml of cBrew. iPSCs were diluted in a pre-differentiation medium, supplemented with Thiazovivin (1:5,000), and seeded in a density of 1.5–2 × 10^5^ cells/cm^2^. The medium was changed on days 2 and 1 to a fresh pre-differentiation medium without Thiazovivin.

For differentiation (d0), pre-differentiating iPSCs were passaged onto PDL/Cultrex-coated plates. Plates were first coated with 20 μg/ml PDL in DBPS 1 day before the start of differentiation (d–1), incubated overnight at 37°C, washed once with DBPS, and then coated with 20 μg/ml Cultrex in DPBS for another hour (d0). Plates were used within 24 h and washed twice with DPBS before seeding the iPSCs.

For seeding, dissociation with Accutase, collection, and centrifugation were performed as described during the pre-differentiation, except for re-suspending the cell pellet in 1 ml of neuronal maturation medium (NMM), instead of using cBrew. Cells were diluted in NMM containing freshly added Geltrex (1:100) to reach a density of 0.5–0.8 × 10^6^ cells/well and 2.5–4 × 10^6^ cells/well in a 24-well plate and 6-well plate or imaging dish, respectively. Half of the amount of NMM was exchanged every weeks (d7, d14, d21) while iPSCs differentiated progressively into iN.

### 2.5 Media recipes/medium ingredients

Pre-differentiation medium:

- KO DMEM/F-12
- 1× neuropan-2 supplement
- 1× non-essential amino acids
- 10 ng/ml BDNF
- 10 ng/ml NT-3
- 1.5 μg/ml laminin
- 2 μg/ml doxycycline
- 1× Antibiotic–antimycotic solution

NMM:

- 50% neurobasal medium
- 50% DMEM/F-12
- 0.5× neuropan-2 supplement
- 1× non-essential amino acids
- 0.5× B27 supplement
- 10 ng/ml BDNF
- 10 ng/ml NT-3
- 1.5 μg/ml laminin
- 2 μg/ml doxycycline
- 1× antibiotic–antimycotic solution

### 2.6 Transfection

SHN were transfected using the polymer-based PolyJet DNA transfection reagent (SignaGen) at d7 of differentiation. Transfection was performed according to the manufacturer’s protocol with the following modifications: prior to transfection, 1/2 of the conditioned medium was collected from each well and stored at 37°C with 5% CO_2_. For 1 well of a 24-well plate, 0.33 μg of plasmid DNA and 1 μl PolyJet reagent were separately mixed with 25 ml DMEM each, then joined, incubated for 10–15 min, and added dropwise to the culture medium. After 3 h, cells were washed once with DMEM and then cultivated in a previously collected medium filled up with fresh SHM-10. The transfection efficiency ranged widely depending on the used plasmid between 5 and 20%.

iN were transfected using the LipoStem transfection reagent (TFS) 2–4 days before experimental use, according to the manufacturer’s protocol. Prior to transfection, 1/2 of the conditioned medium was collected from each well and stored at 37°C with 5% CO_2_. For 1 well of a 24-well plate, 0.5 μg plasmid DNA was mixed with 25 μl of Opti-MEM (mix A) and 0.5 μl LipoStem was mixed with 25 µl DMEM (mix B). Mixes A and B were joined, incubated for 10 min, and added dropwise to the cell culture. After 24 h, cells were washed with warm KO-DMEM and cultivated in a previously collected medium filled up with fresh NMM.

### 2.7 Imaging methods

#### 2.7.1 Immunofluorescence experiments

Cells were fixed with 3.7% formaldehyde (FA) in PBS. After incubation for 18 min, cells were stored in 55% (v/v) glycerol in PBS (storage solution) at −20°C for later analysis or washed three times with PBS for direct staining. For immunofluorescence staining, FA or storage solution was aspired and cells were washed three times in PBS. Cells were permeabilized and blocked in 5% BSA (Carl Roth) and 0.2% Triton X-100 (Carl Roth) in PBS for 10 min. Afterward, cells were washed once in PBS and then stained with primary antibodies at 4°C overnight. On the following day, coverslips were washed three times in PBS and incubated with the secondary antibody for 3 h. Finally, coverslips were washed three times with PBS and stained with Hoechst 33342 (NucBlue™, TFS) in PBS for another 25 min at room temperature. Cells were washed twice with deionized water and then mounted on objective slides with a Poly-Mount mounting medium (Polysciences). Mounted slides were dried for at least 24 h before imaging and kept at 4°C for long-term storage.

Antibodies used were Rabbit Anti-TRIM46 (Synaptic System), Mouse Anti-Ankyrin G (Neuromab), Chicken Anti-MAP2 (Abcam), and Rabbit Anti-TAU (SantaCruz).

#### 2.7.2 Fluorescence microscopy

Microscopy images were taken with a widefield fluorescence microscope (Zeiss), equipped with an LED lamp (Colibri 7, Zeiss) and a fluorescence camera (Axiocam 503 mono, Zeiss). Images were acquired using the Zen imaging software (Blue pro, Zeiss). Images were taken with magnifications of 200×, 400×, or 630×, using 10×, 20×, and 40× objectives air based and the 63× objective with immersion oil (Immersol 518F, Zeiss). Exposure time and laser power were adjusted so that the fluorescence signal was not oversaturated. Within the same experiment, all images were taken with identical LED intensity and exposure time to ensure statistical comparability of the protein expression levels.

#### 2.7.3 Live cell imaging

For investigation of MT dynamics by live imaging, SHN and iN were transfected with a plasmid expressing tdTomato-tagged EB3 (ptdTomato-EB3) following the transfection protocol described above and before [35,36]. SY5Y-cells were transfected on day 7 while iN were transfected on days 12–14 post-differentiation. Generally, the neurons expressed the EB3-tdTomato 2–3 days after transfection. The movement of fluorescently tagged EB3 particles was recorded with a Leica DMi8 S Platform microscope equipped with a Leica-DFC9000 fluorescence camera and the image acquisition software Las X Life Sciences (Leica Microsystems). The cells were continuously covered with a CO_2_ incubation unit and a microscope chamber to protect the cells from external light radiation and were left to accommodate 15 min before imaging. The time-lapse video was composed of images taken at a rate of 1 image per 2 s over a period of 2 min (for in-depth protocols, see also [35]).

### 2.8 qPCR

A quantitative RT-qPCR was performed to assess the expression of TRIM46 and AnkG in different cell types. Cells were harvested and lysed and total RNA was isolated using PureLink RNA Mini Kit (TFS). Reverse transcription into cDNA was carried out with ProtoScript II First Strand cDNA Synthesis Kit (NEB). Quantitative RT-qPCR was performed with SYBR green I (TFS) on the following cycling protocol: denaturation at 95°C for 10 s, annealing at 58.5°C for 30 s, and elongation at 68°C for 60 s, 30 cycles. The HPRT gene was used as a reference gene for relative quantification. Used primers were the following: TRIM46 Primer 1 (GCAGCTGCACAACAGGATTG) and Primer 2 (ATCATAGGCAAAGGTGCGCT); AnkG Primer 1 (GTCTGAGCAAAAGCAGGGAGA) and Primer 2 (ACCGTTCGCTGTTACGAGTG).

### 2.9 Data analysis

#### 2.9.1 EB3 MT analysis

The acquired movie files were registered and exported with LasX Image acquisition software. The movie file was then analyzed with ImageJ/Fiji software (OpenSource) as follows: a 30 μm line region of interest (ROI) was drawn along the proximal axon and the dendrite, respectively, and a 50–200 μm line ROI along the distal axon (at least 400 μm away from soma) with the segmented line tool. The ImageJ plugin Kymograph Reslice Wide generated kymographs from the EB3 comets moving along this linear ROI, which were then read out for (comet number/30 μm/min), comet direction (anterograde/retrograde), and comet speed (μm/min). The comet speed was calculated from the gradient of the comet track in the kymograph. All experiments were conducted in 3 experimental replicates of 20–60 cells each. To compare EB3 dynamics in the different neuronal compartments, it was essential to distinguish axons from dendrites. Axons were distinguished from dendrites using established morphological characteristics (Bell et al., [32]; Tjiang and Zempel, [37]; Zempel et al., [38]), e.g., a constant small diameter, longer outgrowth than dendrites (>300 μm), and branching pattern of 90°. In case of uncertain morphological discrimination, we fixed the neurons after live imaging, re-identified the imaged cells on the gridded imaging chamber, and stained for TAU, MAP2, and AnkG/TRIM46 to ascertain that we analyzed/imaged the appropriate neuronal subcompartments (Figure 3c).

#### 2.9.2 Axonal enrichment factor (AEF)

**Figure 1:**
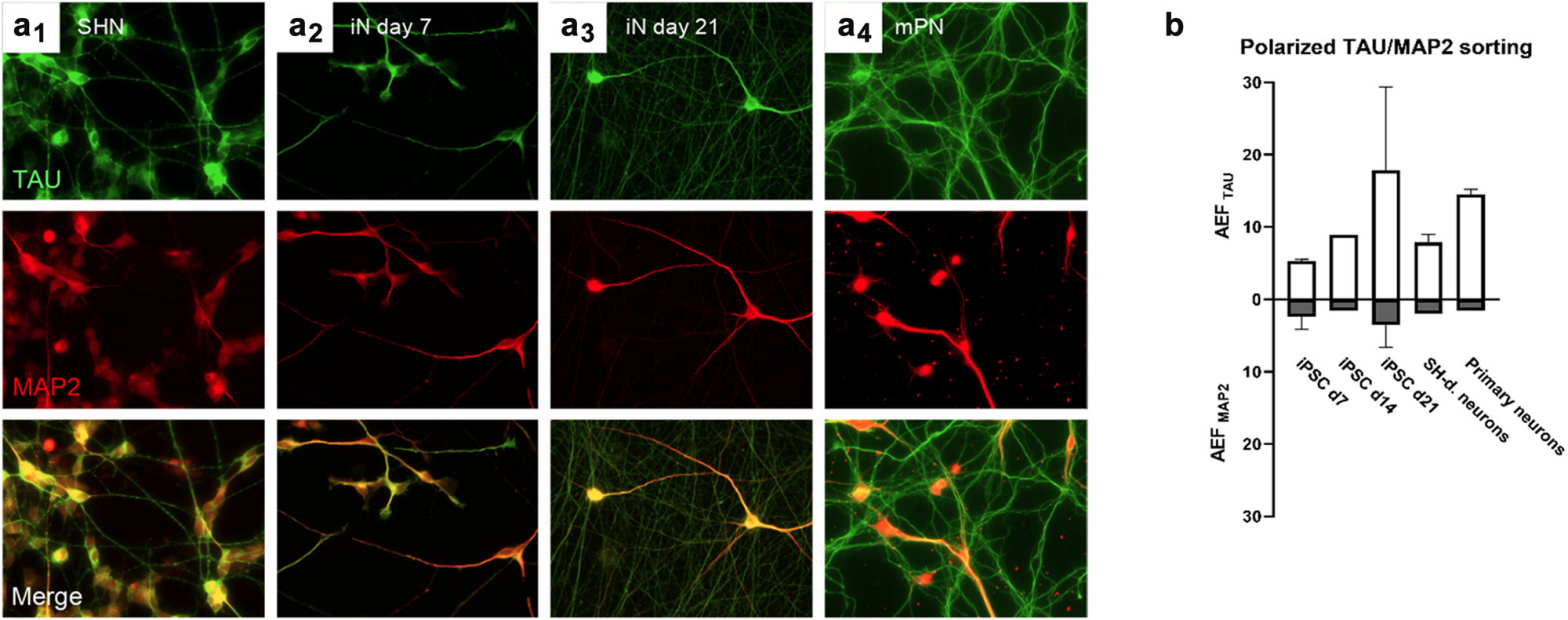
Polarized protein sorting of TAU and MAP2 in different neuronal cell systems: TAU is enriched in the axonal compartment with only little axonal presence of MAP2 in SHN, neurons derived from iPSCs (iN), and primary mouse neurons. Images and quantifications show different types of neurons as indicated stained with antibodies against TAU (K9JA) and MAP2 (chicken polyclonal), and merges. (a1–4) Representative images of SHN (a1), iN at 7 days post-differentiation (d7) (a2), iN at d21 (a3), and primary mouse neurons 9 days *in vitro* (DIV9) (a4), immuno-labeled with TAU (green), and MAP2 (red). (b) Quantification of the AEF of TAU and MAP2 in the investigated neuronal cell types. While MAP2 is only slightly enriched in the axon compared to soma in all cell types, axonal tau enrichment is strongest in iN and increases during maturation from ∼5-fold at d7 to ∼18-fold at d21. Axonal TAU enrichment is equally strong in mPN (∼15-fold) and moderately strong in SHN (∼9-fold, data for SHN obtained from Michael Bell-Simons from Bell et al. [32]). Analysis was done from 3 to 4 experimental replicates with 15–20 neurons each per cell type and age. Standard deviation indicated by error bars upwards (AEF_TAU_) for TAU and downwards (AEF_MAP2_) for MAP2. See Section 2 for a detailed calculation of AEF. Scale bar: 20 µm.

To quantify the enrichment of TAU and MAP2 in the axon, relative to the soma, we calculated an AEF (AEF_Tau_, AEF_MAP2_). For both proteins, we measured the mean fluorescent intensity (MFI) in ROIs drawn in the soma (MFI_S_), the axon (MFI_A_) (>100 µm distal from the AIS), as well as in the empty space next to the soma (MFI_bgS_) and axon (MFI_bgA_), to normalize for background noise. The ROI in the soma was drawn, so it would not overlap with the nuclear signal. We subtracted MFI_S_ – MFI_bgS_ (=MFI_Soma_) and MFI_A_–FI_bgA_ (=MFI_Axon_) to exclude background fluorescent noise. Next, an axon-to-soma ratio was calculated, for TAU and MAP2 each (MFI_Axon_/MFI_Soma_). Finally, to account for volume bias in the fluorescent intensities, we normalized the TAU/MAP2 axon-to-soma ratios to the axon-to-soma ratio of the randomly distributed volume markers tdTomato or GFP \[\left(\phantom{\rule[-0.75em]{}{0ex}},\text{e}\text{.g}\text{.,}\hspace{.25em}{\text{AEF}}_{\text{Tau}}=\frac{{({\text{MFI}}_{\text{Axon}}/{\text{MFI}}_{\text{Soma}})}_{\text{Tau}}}{{({\text{MFI}}_{\text{Axon}}/{\text{MFI}}_{\text{Soma}})}_{\text{tdTomato}}}\right)]\] . ROIs for tdTomato, GFP, MAP2, and TAU were identical. Quantitative data for AEF of SHN from Figure 1b (fourth bar of quantification) was obtained from Michael Bell-Simons from Bell et al. [32] and replotted for comparison.

#### 2.9.3 AnkG/TRIM46 enrichment factor

**Figure 2:**
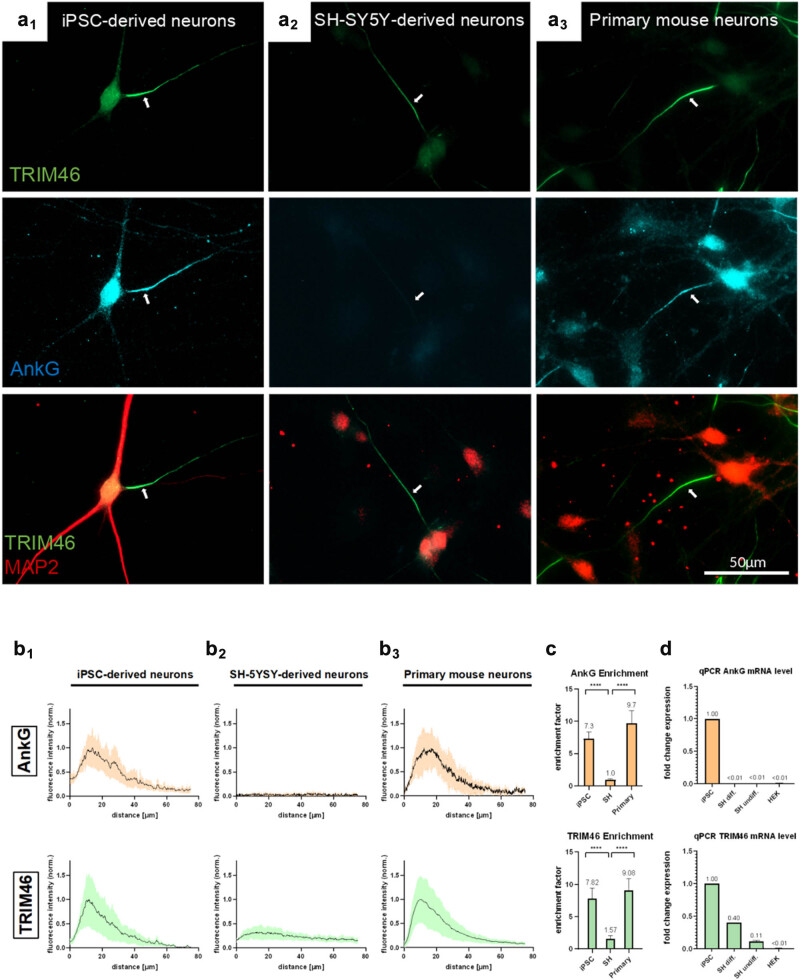
AIS development in different neuronal cell systems shows classic AIS structure in iN and primary mouse neurons with marked AnkG and TRIM46 enrichment, in contrast to SHN with AnkG deficiency and moderate TRIM46 enrichment in the proximal axon. (a1–3) Representative images of iN at day 17 post-differentiation (d17) (a1), differentiated SHN (a2) and primary mouse neurons (mPN), 9 days *in vitro* (DIV9) (a3), immuno-labeled with AnkG (blue) and TRIM46 (green) and MAP2 (red) antibody. Note clear AIS staining in the proximal axon of iN and mPN, but absent AnkG staining and moderate TRIM46 staining in SHN. (b1–3) Fluorescence profiles of AnkG and TRIM46 staining in the proximal axon. Note that absolute values were normalized to a maximum of iN plot (b1, b2), or the maximum of primary neurons plot (b3). Data from B2 and B3 were obtained from Michael Bell-Simons for comparison [32]. The AIS profile appears typical for both AIS proteins in iN and mPN. In contrast, relatively low TRIM46 levels and negligible antibody labeling for AnkG in SHN. (c) AIS enrichment factor for AnkG (orange) and TRIM46 (green) in the three cell types. TRIM46 is much less locally enriched (∼1.6 fold) in SHN than in the other neuronal cell models. (d) qPCR of AnkG and TRIM46 mRNA levels reveal no AnkG expression, but increasing TRIM46 expression during differentiation in SH-SY5Y cells. Statistics: (b1–3) Analysis was done from 3 to 4 experimental replicates with 15–20 neurons each per cell type with a two-way analysis of variance with post hoc Sidak’s multiple comparisons. The alpha level is 0.05, and variance is between groups. Significance levels: *p < 0.05, **p < 0.01, ***p < 0.001. Standard deviation is indicated by area fill around line (b) or error bars (c). (d) Analysis was done from two experimental replicates per cell type with 3 technical replicates each.

To quantify the localized enrichment of TRIM6 and AnkG (AIS-EF_TRIM46_, AIS-EF_AnkG_) at the beginning of the axon, a ratio between the peak fluorescence intensity (all values >70% of maximum value) and the baseline fluorescence intensities along the AIS (first 80 μm) was calculated. The peak intensity (*y*) was determined as the mean value of all values greater than 70% of the maximum fluorescence signal, along the peak zone of a certain length (*x*). The baseline intensity was determined as the mean value of all fluorescence intensities over the same distance of (*x*) μm at the end of the 80 μm ROI. AIS enrichment factor (AIS EF) was calculated from three experimental replicates for all cell types. Quantitative data for AIS plot profile (Figure 2b2, 3) and AIS enrichment factor of SHN and mPN (Figure 2c, second and third bars) were obtained from Michael Bell-Simons from Bell et al. [32] and replotted for comparison.

## 3 Results

### 3.1 Successful establishment of neuronal cell polarity and TAU distribution in human neurons *in vitro*

In AD and related neurodegenerative tauopathies (neurodegenerative diseases [NDD]/tauopathy), cell polarity is impaired, reflected by TAU mislocalization into the somatodendritic compartment [39,40]. To have a better understanding of the cell culture systems, we first tested which human neuronal cell model is able to recapitulate neuronal cell polarity in terms of successful axonal targeting of TAU and somatodendritic retention of MAP2 similar to primary rodent neurons.

We differentiated SH-SY5Y cells to neurons (SHN) using our established protocol, which resulted in a relatively high yield of differentiated cells (∼75%), successful axodendritic polarization, and convincing TAU enrichment in the axon after 14 days of differentiation using RA and BDNF, as detailed previously [32,33]. We also differentiated our iN as established previously using doxycycline-dependent expression of Ngn2, resulting in a differentiation efficiency of >90%, axodendritic polarization, and successful TAU sorting [26,27] (Figure S1). Finally, we used the established standard model of mammalian neurobiology, mouse primary forebrain neurons, which shows excellent axonal targeting of endogenous TAU and even allows to discriminate subtle differences of axodendritic targeting of, e.g., different isoforms of pseudophosphorylated TAU [34,38,41].

We found that all neuron types investigated, after sufficient differentiation as appropriate and necessary (i.e., 14 days for human neurons, >7 days for murine neurons, for details, see [33,34]), developed axons and dendrites. When stained for the axonal marker TAU and the somatodendritic marker MAP2 and subsequently imaged via immunofluorescence microscopy (IF), we found that TAU was strongly enriched in the axons, but not MAP2, which remained largely in the soma and the dendrites (Figure 1a). Morphologically, dendrites were larger, both in diameter and length, in iN and primary neurons compared to SHN (Figure 1a). Quantification of the AEF, a measure of enrichment of a protein in the axon compared to the soma (see Section 2 for details) revealed that in all cell types, MAP2 is only slightly enriched in the axon compared to the soma, while TAU is distinctly enriched in the axon. Axonal TAU enrichment is strongest in iN and increases during maturation from ∼5-fold at day 7 after differentiation (d7) to ∼18-fold at d21. Axonal TAU enrichment is equally strong in mPN (∼15-fold) and moderately strong in SHN (∼9-fold). Nonetheless, this means that both SHN and iN show strong axonal enrichment of TAU, but not of MAP2, comparable to murine primary neurons, indicative of successful axodendritic development and establishment of neuronal cell polarity.

### 3.2 Pronounced enrichment of AnkG and TRIM46 in iN, but not in SH-SY5Y neurons

Next, we compared the AIS development in three neuronal cell systems: primary mouse neurons, iN, and SHN. We conducted AnkG and TRIM46 immunostainings and measured the fluorescent intensities, localization, and local enrichment using IF and calculation of the AIS enrichment factor (AIS-EF), as well as their mRNA amounts via qPCR. Both iN and primary neurons showed pronounced expression of AnkG as well as TRIM46 along the proximal axon and displayed the typical morphology of an AIS in IF (Figure 2). The length and location of their AIS, situated approximately 5–40 μm distal to the soma in the proximal axon, were identical: The proximal enrichment of AnkG (mPN: 9.7-fold, iPSC: 7.3-fold) and TRIM46 (mPN: 9.1-fold, iN: 7.8-fold) was pronounced in both mPN of age DIV9 and in iN at day 17 of differentiation. Moreover, the maximal AnkG and TRIM46 fluorescence were situated ±10 μm distally from the soma and had the same spatial distance of ±2 μm from each other in both cell models (Figure 2b; TRIM46 max. at 10.4 µm, AnkG max. at. 12.4 µm in mPN, TRIM46 max. at 11.8 μm, AnkG max. at. 13.8 μm in iN). In contrast, IF showed no immuno-labeling of AnkG in SHN. Nevertheless, TRIM46 was expressed in this cell line after differentiation, however, relatively moderate (Figure 2a2 and 2b2) in comparison to iN. Moreover, the local enrichment of TRIM46 (1.6-fold) was much less pronounced than in iN (7.8-fold) and primary neurons (9.1-fold) (Figure 2a and b, quantifications in Figure 2c). Secondary antibody control showed no unspecific binding (Figure S2).

To confirm the lower expression level of the AIS-related proteins in SHN, we conducted qPCR to assess the mRNA levels of TRIM46 and AnkG in iN, differentiated SHN, undifferentiated naive SH-SY5Y cells, and HEK cells. The HEK cells served as a negative control for qPCR since no expression of AIS proteins is expected in these cells derived from human embryonic kidney cells. In line with the findings from the IF, there was no expression of AnkG in undifferentiated, naive SH-SY5Y cells (<1% compared to iN). But notably, also AnkG mRNA levels in differentiated SHN were negligible (<1% compared to iN). However, we detected expression of TRIM46 in SHN, which augmented from 10.7% (naive) to 39.9% (differentiated neurons, both values compared to iN) during their differentiation, which is consistent with the moderate signal of TRIM46 in the immunostainings. As expected, we found no expression (<1% of iN) of neither AnkG nor TRIM46 in HEK cells (*n* = 1–2 experiments).

Taken together, these data demonstrate that iN develop an AIS, morphologically alike of that in primary mouse neurons. On the other hand, SHN only express TRIM46 in the axon upon neuronal differentiation but lack AnkG, which likely results in a differently organized AIS structure.

### 3.3 Anterograde MT orientation in axons of both iN and SHN, more retrograde MT orientation in iN dendrites, growth events in SHN

After observing the absence of a classic AIS, in terms of no detected AnkG expression and low TRIM46 levels, in SHN compared to iN, we investigated if this influences the MT dynamics, which are of great importance for neuronal polarization as well as the pathophysiology of AD and NDD in humans.

**Figure 3:**
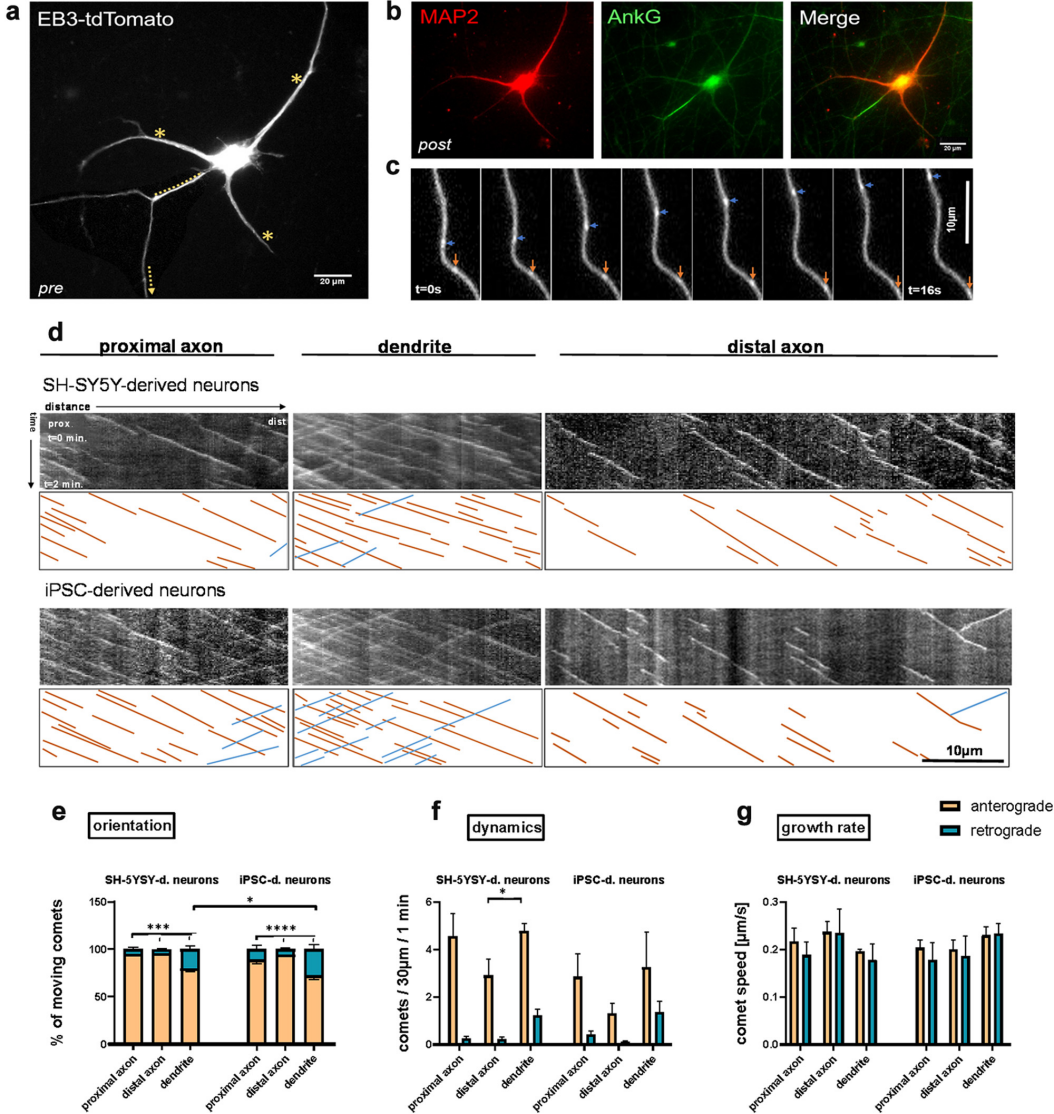
Similar speed of MT polymerization in both SHN and iN, but slightly more retrograde MT growth events in iN. Comparison of MT organization between proximal axon, distal axon, and dendrite in SHN and iN at days 14–18 of differentiation (d14–d18) by EB3 Live Imaging show axodendritic MT polarity. (a) Representative image of an iN transfected with pEB3-tdtomato. Dashed lines indicate the regions where EB3 comets were imaged within the neuron: proximal axon, dendrite, and distal axon (outside of image frame, at least 400 μm distal from soma). (b) Post-imaging: Representative image of a neuron fixed and immunolabeled with AnkG and MAP2 antibody after video recordings (see a). (c) Representative close-up time frame pictures of two oppositely moving EB3 comets (orange arrowhead = anterograde, blue arrowhead = retrograde) in the distal axon of an iN. (d) Representative kymographs derived from recordings of all three compartments in SHN and iN. The *y*-axis corresponds to time. The *x*-axis corresponds to the traveled distance. Upper row of kymographs for SHN and iN: original in B/W. Lower row of kymographs for SHN and iN: illustrative highlighting of comet trails declining lines (orange) represent anterograde comets and inclining lines (blue) represent retrograde comets. Scale bar: 10 μm. (e–g) Orientation, number, and growth rate of anterograde (orange) and retrograde (blue) comets in SHN and iN. (e) Comet direction indicates MT orientation. Note the higher proportion of retrograde comets in the dendritic compartment. (f) Number of moving comets represents a dynamic property of MTs. Note more growth events in the proximal compartments and in SH-SY5Y-d neurons. (g) Speed of moving comets represents the MT growth rate. Overall little deviation from the average growth rate of 12.5 μm/min. Statistics: a two-way analysis of variance and Tukey’s correction for multiple comparisons were performed to determine significance in (e) and (f). Significance levels: *p < 0.05, **p < 0.01, ***p < 0.001. (e) Error bars of the anterograde portion pointing downwards, and error bars of the retrograde portion pointing upwards.

To investigate the dynamics and orientation of MTs in different compartments and cell types, we transfected both our human SHN and human iN with tdTomato-tagged EB3 on day 7, respectively, days 12–14 (Figure 3a–d) (Seq. S1). The protein EB3 binds to the plus tips of growing MTs and, when conjugated with a fluorescent protein like tdTomato, enables live-tracing of MT growth events on MT plus ends (Figure 3b). These moving EB3 accumulations appear as comet-like structures in the time-lapse recordings, whose direction, speed, and quantity can be read out from kymographs, generated from the time-lapse videos (Figure 3d) (Movie S1). Kymographs are imaging tools that visualize dynamic processes in biological systems by capturing time-lapse images along a specified axis. Each pixel represents a position and time, creating a two-dimensional graph where the *x*-axis denotes space and the *y*-axis indicates time. This technique is particularly effective for studying the movement of cellular structures along cytoskeletal elements, revealing insights into transport mechanisms and motility. We recorded and analyzed EB3 movements in the proximal axon, distal axon, and dendrites of the neurons on days 14–18 of differentiation when cell polarity is well-established. We used morphological characteristics and/or post-imaging immunostaining to distinguish axons from dendrites (see Section 2.9.1). While in both cell types identification of the axon is relatively simple (as the axon is smaller and constant in diameter, there are different branching patterns of axons and dendrites, and very different lengths of axons and dendrites as described before [32,37,38], we nonetheless fixed the cells, re-identified the imaged cells, and stained for TAU, MAP2, and ANKG/TRIM46 in initial experiments to ascertain that we analyzed/imaged the appropriate neuronal subcompartments (Figure 3c).

Counting the EB3 comets, we found that in all cell compartments and in both cell types, the majority of MTs was orientated anterogradely (thus with the growing plus end directed to the periphery), represented by EB3 moving outwards from the soma; however, there were notable differences: In SHN, the proportion of anterograde comets was equally high in the proximal axon (94.9% ± 2.0) and distal axon (95.8% ± 0.8), whereas the proportion of anterograde comets was lower in the dendrites (79.8% ± 3.6). Consequently, the percentage of retrograde comets was ∼4–5 times higher in the dendrites (20.2% ± 3.6) than in the axonal compartments (proximal: 5.1% ± 2.0, distal: 4.2 ± 0.8%). A similar distribution was observed in iN, where anterograde comets accounted for 89.0% ± 4.1 in the proximal and 94.3% ± 1.3 in the distal axon of all moving comets. However, the proportion of anterograde comets in the dendrites (72.4 ± 3.4%) was even lower than in the dendrites of SHN, resulting in a significantly higher portion of retrograde comets (27.6% ± 4.7) compared to the other compartments and cell types (Figure 3d and e).

We also quantified the number of moving comets (comet number/30 μm/min), representing the amount of dynamic MTs and thus the overall growth dynamics (Figure 3f). Generally, in both cell models, the highest amount of total moving comets was observed in the dendrites (SHN: 6.0 ± 0.4, iN: 6.1 ± 2.6), followed by those in the proximal axon (SHN: 4.8 ± 1.0, iN: 4.3 ± 1.5) whereas the number of moving comets in the distal axon was significantly lower (SHN: 3.1 ± 0.7, iN: 1.9 ± 0.6). Strikingly, the number of moving comets in the SHN exceeded those in the iN in all three compartments for anterogradely moving comets, but not for retrogradely moving comets.

Finally, we analyzed the speed of the moving comets from the gradient of the comet traces within the kymographs (Figure 3g). The average speed of all moving comets was 12.9 ± 1.1 μm/min in SHN and 12.1 ± 1.1 μm/min in iN. Broadly, the average speed was similar between the two cell types and also between the different compartments, with differences of only 1–2 μm/min. In the SHN, comets moved fastest in the distal axon (14.1 ± 2.1 μm/min), whereas comets of iN had the highest speed (13.9 ± 1.1 μm/min) in the dendrites. Interestingly, the speed of anterograde comets was always ∼1–2 µm/min higher than the retrograde ones in both cell types with the exception of the dendritic compartment in iN, where retrograde comets moved faster than the anterograde ones. However, the differences appeared overall marginal and not statistically significant.

In summary, MTs in SHN and iN behaved remarkably similarly regarding orientation, dynamics, and speed of growth, but with a higher proportion of retrogradely moving comets particularly in iN dendrites.

## 4 Discussion

### 4.1 Neurons acquire axon formation and polarized TAU sorting without AnkG-organized AIS

In this study, we set out to evaluate whether and which type of polarized human neurons would be a suitable cell model to investigate compartment-specific MT dynamics, in order to test human model systems also for future studies in the context of axodendritic TAU (mis-)sorting, AD, and associated tauopathies/neurodegeneration (NDD). We used the SH-SY5Y cell line and neurons derived thereof (SHN), which are cost-efficient and which we had judged to be potentially suitable for TAU-based research [30,33], and iPSC that can be differentiated via doxycycline-induced expression of Ngn2 into forebrain neurons (iN), which are also valuable for studying, e.g., neuronal activity in disease settings, all of which we have described previously [27,42]. We used as a reference the standard model of cellular neurobiology, mouse primary neurons (mPN) [36,43].

We assessed if the three neuronal models acquired neuronal polarity, which includes the structural separation of axons and dendrites with compartment-specific enrichment of MAP2 in dendrites and TAU in axons (Figure 1). Our findings indicate that both SHN and iN models show strong axonal enrichment of TAU, comparable to murine primary neurons. Specifically, axonal TAU enrichment was strongest in iN, increasing from approximately 5-fold at day 7 to 18-fold at day 21 post-differentiation. This enrichment was equally strong in mPN (∼15-fold) and moderately strong in SHN (∼9-fold). These results suggest that both SHN and iN are suitable models for studying TAU sorting and neuronal polarity, with iN showing the highest degree of TAU enrichment.

Strikingly, architectural properties of axons and dendrites were obtained and axonal outgrowth occurred extensively not only in the AIS-positive iN and primary neurons but also in the AnkG-deficient SHN. Additionally, immunostainings for MAP2 and TAU revealed a clearly polarized distribution of the two proteins in all cell types. We also compared AIS development in iN, primary mouse neurons, and SHN, whereas the AIS composition was much alike in primary neurons and iN, we found that SHN did not express AnkG, but only TRIM46 in moderate amounts (Figure 2). Importantly, TRIM46 expression augmented during differentiation, which is in line with its reported role in initial neuronal polarization [18,44]. AnkG was reported to maintain neuronal polarity and gate the axonal identity [45,46,47], but SHN showed both axon formation as well as polarized protein sorting despite AnkG deficiency. This is in line with AnkG as the sole actor governing the axonal identity being questioned in various studies [18,32,48]. So what is the interaction and the role of TRIM46 and AnkG?

### 4.2 Functional crosstalk between AnkG and TRIM46

We found that while AnkG and TRIM46 are both involved in establishing neuronal polarity, they play distinct roles. TRIM46 appears to act earlier to initiate axon formation and polarized protein sorting, while AnkG is important for maintaining the axon-dendrite separation later in development [18]. Several lines of evidence suggest that TRIM46 can localize and function independently of AnkG at the axon initial segment (AIS): TRIM46 clusters at the proximal axon earlier than AnkG during development, with 70% of neurites in stage 2 neurons being TRIM46+/AnkG− [49]. In dorsal root ganglion (DRG) neurons that lack AnkG, TRIM46 is still confined to the proximal axon. AnkG knockdown in young hippocampal neurons caused only moderate mislocalization of TRIM46 from the proximal axon [50,51]. Treating mature hippocampal neurons with AnkG shRNA for over a week reduced TRIM46 levels by only 30% [52]. This suggests that TRIM46 may be recruited to the AIS by binding partners other than known AIS components like ankyrin, spectrins, or sodium channels. The data indicate that TRIM46 can autonomously localize to establish the initial MT organization and polarity at the nascent AIS, independent of AnkG.

A key finding is that axonal TAU enrichment correlates with the uniform MT orientation established by TRIM46, even in AnkG-deficient SHN. This could suggest that TAU axonal sorting depends on the MT lattice organization rather than a physical diffusion barrier at the AIS, as proposed previously [53]. Yet, TRIM46 may be sufficient to generate this polarized MT array conducive for axonal TAU sorting, independent of AnkG.

### 4.3 MT dynamics and impact of MT orientation on TAU sorting

MT dynamics, crucial for neuronal polarization and function, were investigated using EB3-comet tracking. Both SHN and iN showed an axodendritic polarity in MT orientation: while MTs were orientated anterogradely in 90–95% in axons of both SHN and iN, dendrites showed MTs of mixed polarity with only 70% (iN) to 80% (SHN) of MT plus-ends directed anterogradely. Importantly, this polarized organization occurred independently of AnkG, which is absent in SHN, supporting the hypothesis that TRIM46 is sufficient to establish axonal MT polarity [18,54]. The extent of MT reorientation from mixed/multipolar in stage 2 neurons to uniform in stage 3 axons matched previous reports in rodent neurons [55]. Recent investigations in iN displayed a similar trend: in unpolarized stage 2 neurons, 80% of MTs had anterograde orientation which increased to 90% in axons of stage 3 neurons. The fraction of plus-end-out MTs in the dendrites decreased from 80% to 60% in stage 3 neurons [19]. The higher proportion of retrograde dendritic MTs in iN in comparison to SHN could thus hint towards a higher grade of maturity, consistent with structural differences between the two neuronal cultures. Nevertheless, both SH-SY5Y and iN show the MT cytoskeleton of a polarized neuron.

Can we now hypothesize that axonal TAU sorting depends on uniform MT orientation in the axon? Indeed, polarized protein sorting correlated with MT directionality in iN and SHN. In both neurons, MAP2 accumulated in regions with mixed MT polarity, whereas TAU was enriched in axons with unidirectional MTs. Consistently, TAU was distributed evenly throughout unpolarized stage 2 iN (correlating well to our d7 iNs), which have multipolar MTs in their developing neurites [19]. Hence, axonal MT orientation may drive anterograde TAU mobility, e.g., as suggested previously by kinesin motors [56]. Additionally, MT properties – orientation, PTM, spacing – in the AIS may enforce TAU binding and thereby a diffusional gradient of soluble TAU from the soma to the axon [57]. Contrarily, MAP2 might be unable to couple to axonal MT motors, as described for other somatodendritic cargo [58], and therefore be hindered to drive along axonal MTs. It is thus possible that differential MT orientation in axons and dendrites drives polarized protein sorting by selective acquisition of motors. These results would be compatible both with TAU being simply an MT binder, but also with a functional role of TAU modifying MT liability as proposed previously [59].

### 4.4 Differences in MT dynamics in different model systems

Interestingly, SHN exhibited more growth events in all compartments compared to iN, indicating higher overall MT dynamics. This might be due to a higher immaturity of SHN. The speed of EB3 comets was consistent among the compartments as well as among the two cell models, with an average speed of 12.1 μm/min in SHN and 12.9 μm/s in iN. Anterograde growth events were slightly faster than retrograde, except for dendritic MTs in iN. Brown-Handerson et al. reviewed MAPs that regulate MT growth, i.e., catastrophe frequency, and rescue frequency and also growth speed. The differential composition of accelerating MAPs on plus and minus tips may result in a faster growth rate of anterograde MTs. Indeed, Jakobs et al. propose that faster polymerization of anterograde MTs contributes to uniform MT orientation in the axon [60]. Nevertheless, as the polymerization rate is not significantly faster in axons than in dendrites (here and elsewhere [19,60,61]), the extensive outgrowth and specification of the axon likely results from differential MT orientation, reduced catastrophe frequency, or increased rescue frequency – as mediated by for TRIM46 [44] – and not by faster growth rates. Consistently, silencing of TRIM46 does not decrease the MT polymerization rate but the portion of anterograde MTs and thereby axonal outgrowth [62].

Interestingly, growth speed in human neurons was substantially faster than in numerous other neurons observed in former studies, e.g., 0.12 μm/s (7 μm/min) in *Drosophila* dendrites [63], 5 μm/min in axons of *Drosophila* [60], 0.1 μm/s (6 μm/min) in *Caenorhabditis elegans* [64], 0.1 μm/s (6 μm/min) in cortical mouse neurons [16], or 0.08 μm/s (4.8 μm/min) [62], respectively, and 0.2–0.3 μm/s (13–19 μm/min) in hippocampal rat cultures [61]. Importantly, the growth rate appears not related to the fluorescent + TIP used. Thus, these differences could hint toward species-dependent regulation of MT growth: possibly, MT polymerization is regulated faster in humans, since MTs have to accomplish a much longer neurite growth than in neurons of rodents or non-vertebrates.

Additionally, we investigated the absolute number of MT growth events within a defined distance of 30 µm along the neurites. Both in SHN and iN, we observed most MT growth events in the dendrites, followed by the proximal axon, and least in the distal axon (Figure 3). This can be explained either by a higher density of MT in the dendrites or alternatively by a greater number of dynamic MTs compared to stable MTs in dendrites, as reported before [19].

How neurons establish a polarized MT network is a broadly debated question in neurobiology. Likely a combination of parallel bundling by TRIM46, local MT nucleation sites, translocation of anterograde and retrograde MTs, de novo MT generation from pre-existing MTs, differential growth behaviors of anterograde and retrograde MTs controlled by associated MAPs, is responsible for unipolar MTs in axons. We can now hypothesize that besides axonal outgrowth and neuronal transport, polarized MT orientation is essential for axonal TAU sorting, but further studies are needed to elucidate the regulators of MT polymerization and its impact on TAU sorting in humans.

## 5 Conclusion

In sum, in this study, we show that while SHN compared to iN lack a proper AIS as defined by the presence of AnkG, axonal targeting of TAU and axodendritic MT polarization is comparable, making both human neuron cell culture types suitable for studying neuronal cell polarity and further understanding of MT-related features. This holds true in particular for SHN, if the focus is *not* the AIS. Finally, to assess both the influence of the AIS and MT-associated factors on TAU sorting, iN are the more appropriate model to gain a holistic understanding of pathomechanisms in AD/NDD and associated dysfunction of neuronal cell polarity.

## Supplementary Material

Supplementary material

## References

[1] Zempel H. Genetic and sporadic forms of tauopathies—TAU as a disease driver for the majority of patients but the minority of tauopathies. Cytoskeleton. 2024;81(1):66–70. 10.1002/cm.21793 37795931

[2] Langerscheidt F, Wied T, Al Kabbani MA, van Eimeren T, Wunderlich G, Zempel H. Genetic forms of tauopathies: inherited causes and implications of Alzheimer’s disease-like TAU pathology in primary and secondary tauopathies. J Neurol. 2024;271(6):2992–3018. 10.1007/s00415-024-12314-3 PMC11136742 38554150

[3] Zempel H, Mandelkow EM. Linking amyloid-β and Tau: Amyloid-β induced synaptic dysfunction via local wreckage of the neuronal cytoskeleton. Neurodegener Dis. 2012;10(1–4):64–72. 10.1159/000332816 22156588

[4] Zempel H, Luedtke J, Kumar Y, Biernat J, Dawson H, Mandelkow E, et al. Amyloid-β oligomers induce synaptic damage via Tau-dependent microtubule severing by TTLL6 and spastin. EMBO J. 2013;32(22):2920–37. 10.1038/emboj.2013.207 PMC3831312 24065130

[5] Zempel H, Mandelkow EM. Tau missorting and spastin-induced microtubule disruption in neurodegeneration: Alzheimer disease and hereditary spastic paraplegia. Mol Neurodegener. 2015 Dec;10(1):68. 10.1186/s13024-015-0064-1 PMC4687341 26691836

[6] Rogowski K, Hached K, Crozet C, van der Laan S. Tubulin modifying enzymes as target for the treatment oftau-related diseases. Pharmacol Ther. 2021;218:107681. 10.1016/j.pharmthera.2020.107681 32961263

[7] Leterrier C, Dubey P, Roy S. The nano-architecture of the axonal cytoskeleton. Nat Rev Neurosci. 2017;18:713–26. 10.1038/nrn.2017.129 29097785

[8] Lasser M, Tiber J, Lowery LA. The role of the microtubule cytoskeleton in neurodevelopmental disorders. Front Cell Neurosci Front Media SA. 2018;12:165. 10.3389/fncel.2018.00165 PMC6010848 29962938

[9] Mencer S, Kartawy M, Lendenfeld F, Soluh H, Tripathi MK, Khaliulin I, et al. Proteomics of autism and Alzheimer’s mouse models reveal common alterations in mTOR signaling pathway. Transl Psychiatry. 2021;11(1):480. 10.1038/s41398-021-01578-2 PMC8448888 34535637

[10] Tripathi MK, Kartawy M, Ginzburg S, Amal H. Arsenic alters nitric oxide signaling similar to autism spectrum disorder and Alzheimer’s disease-associated mutations. Transl Psychiatry. 2022;12(1):127. 10.1038/s41398-022-01890-5 PMC8964747 35351881

[11] Conde C, Caceres A. Microtubule assembly, organization and dynamics in axons and dendrites. Nat Rev Neurosci. 2009;10(5):319–32. 10.1038/nrn2631 19377501

[12] Kapitein LC, Hoogenraad CC. Building the neuronal microtubule cytoskeleton. In Neuron. Vol. 87, Cell Press; 2015. p. 492–506 10.1016/j.neuron.2015.05.046 26247859

[13] Sánchez-Huertas C, Freixo F, Viais R, Lacasa C, Soriano E, Lüders J. Non-centrosomal nucleation mediated by augmin organizes microtubules in post-mitotic neurons and controls axonal microtubule polarity. Nat Commun. 2016;7:12187. 10.1038/ncomms12187 PMC4947180 27405868

[14] Akhmanova A, Hoogenraad CC. Microtubule minus-end-targeting proteins. Current Biol. 2015;25:R162–71. 10.1016/j.cub.2014.12.027 25689915

[15] Baas PW, Lin S. Hooks and comets: The story of microtubule polarity orientation in the neuron. Dev Neurobiol. 2011 May;71(6):403–18. 10.1002/dneu.20818 PMC3151545 21557497

[16] Kleele T, Marinković P, Williams PR, Stern S, Weigand EE, Engerer P, et al. An assay to image neuronal microtubule dynamics in mice. Nat Commun. 2014;5:4827. 10.1038/ncomms5827 PMC4175586 25219969

[17] Nguyen MM, Stone MC, Rolls MM. Microtubules are organized independently of the centrosome in Drosophila neurons. Neural Dev. 2011;6(1):1–16. 10.1186/1749-8104-6-38 PMC3271965 22145670

[18] Van Beuningen SFB, Will L, Harterink M, Chazeau A, Van Battum EY, Frias CP, et al. TRIM46 controls neuronal polarity and axon specification by driving the formation of parallel microtubule arrays. Neuron. 2015;88(6):1208–26. 10.1016/j.neuron.2015.11.012 26671463

[19] Lindhout FW, Kooistra R, Portegies S, Herstel LJ, Stucchi R, Snoek BL, et al. Quantitative mapping of transcriptome and proteome dynamics during polarization of human ipsc-derived neurons. Elife. 2020;9:1–25. 10.7554/eLife.58124 PMC7498259 32940601

[20] Kapitein LC, Yau KW, Gouveia SM, van der Zwan WA, Wulf PS, Keijzer N, et al. NMDA receptor activation suppresses microtubule growth and spine entry. J Neurosci. 2011;31(22):8194–209. 10.1523/JNEUROSCI.6215-10.2011 PMC6622869 21632941

[21] Nakata T, Hirokawa N. Microtubules provide directional cues for polarized axonal transport through interaction with kinesin motor head. J Cell Biol. 2003;162(6):1045–55. 10.1083/jcb.200302175 PMC2172855 12975348

[22] Buchholz S, Zempel H. The six brain-specific TAU isoforms and their role in Alzheimer’s disease and related neurodegenerative dementia syndromes. Alzheimer’s Dement. 2024;20(5):3606–28. 10.1002/alz.13784 PMC11095451 38556838

[23] Ichida JK, Kiskinis E. Probing disorders of the nervous system using reprogramming approaches. EMBO J. 2015;34(11):1456–77. 10.15252/embj.201591267 PMC4474524 25925386

[24] Mertens J, Marchetto MC, Bardy C, Gage FH. Evaluating cell reprogramming, differentiation and conversion technologies in neuroscience. Nat Rev Neurosci. 2016;17(7):424–37. 10.1038/nrn.2016.46 PMC6276815 27194476

[25] Tripathi MK, Ojha SK, Kartawy M, Hamoudi W, Choudhary A, Stern S, et al. The NO answer for autism spectrum disorder. Adv Sci. 2023;10(22):2205783. 10.1002/advs.202205783 PMC10401098 37212048

[26] Wang C, Ward ME, Chen R, Liu K, Tracy TE, Chen X, et al. Scalable production of iPSC-derived human neurons to identify tau-lowering compounds by high-content screening. Stem Cell Rep. 2017 May;9(4):1221–33. 10.1016/j.stemcr.2017.08.019 PMC5639430 28966121

[27] Buchholz S, Bell-Simons M, Cakmak C, Klimek J, Gan L, Zempel H. Cultivation, differentiation, and lentiviral transduction of human-induced pluripotent stem cell (hiPSC)-derived glutamatergic neurons for studying human Tau. Methods Mol Biol. 2024;2754:533–49. 10.1007/978-1-0716-3629-9_31 38512688

[28] Hamoudi W, Tripathi MK, Ojha SK, Amal H. A cross-talk between nitric oxide and the glutamatergic system in a Shank3 mouse model of autism. In: Free Radical Biology and Medicine. Elsevier; 2022. p. 83–91. 10.1016/j.freeradbiomed.2022.06.007 35716826

[29] Bell M, Zempel H. A simple human cell model for TAU trafficking and tauopathy-related TAU pathology. Neural Regener Res. 2022;17:770–2. 10.4103/1673-5374.322450 PMC8530135 34472464

[30] Bell M, Zempel H. SH-SY5Y-derived neurons: A human neuronal model system for investigating TAU sorting and neuronal subtype-specific TAU vulnerability. Rev Neurosci. 2022 May;33(1):1–15. 10.1515/revneuro-2020-0152 33866701

[31] Kovalevich J, Langford D. Considerations for the use of SH-SY5Y neuroblastoma cells in neurobiology. Methods Mol Biol. 2013;1078:9–21. 10.1007/978-1-62703-640-5_2 PMC5127451 23975817

[32] Bell M, Bachmann S, Klimek J, Langerscheidt F, Zempel H. Axonal TAU sorting requires the C-terminus of TAU but is independent of ANKG and TRIM46 enrichment at the AIS. Neuroscience. 2021;461:155–71. 10.1016/j.neuroscience.2021.01.041 33556457

[33] Langerscheidt F, Bell-Simons M, Zempel H. Differentiating SH-SY5Y cells into polarized human neurons for studying endogenous and exogenous Tau trafficking: Four protocols to obtain neurons with noradrenergic, dopaminergic, and cholinergic properties. Methods Mol Biol. 2024;2754:521–32. 10.1007/978-1-0716-3629-9_30 38512687

[34] Buchholz S, Bell-Simons M, Haag N, Zempel H. Tracking Tau in neurons: How to grow, fix, and stain primary neurons for the investigation of Tau in all developmental stages. In: Smet-Nocca C, editor. Tau protein: Methods and protocols. New York, NY: Springer US; 2024. p. 507–19. 10.1007/978-1-0716-3629-9_29. 38512686

[35] Allroggen N, Breuer H, Bachmann S, Bell M, Zempel H. Studying microtubule dynamics in human neurons: Two-dimensional microtubule tracing and kymographs in iPSC- and SH-SY5Y-derived neurons for Tau research. Methods Mol Biol. 2024;2754:561–80. 10.1007/978-1-0716-3629-9_33 38512690

[36] Zempel H, Mandelkow EM. Tracking Tau in neurons: How to grow, fix, and stain primary neurons for the investigation of Tau in all developmental stages. In: Methods in Molecular Biology. New York: Humana Press; 2017. p. 327–34. 10.1007/978-1-4939-6598-4_20 27975260

[37] Tjiang N, Zempel H. A mitochondria cluster at the proximal axon initial segment controls axodendritic TAU trafficking in rodent primary and human iPSC-derived neurons. Cell Mol Life Sci [Internet]. 2022;79(2):120. 10.1007/s00018-022-04150-3. PMC8816743 35119496

[38] Zempel H, Dennissen FJA, Kumar Y, Luedtke J, Biernat J, Mandelkow EM, et al. Axodendritic sorting and pathological missorting of Tau are isoform-specific and determined by axon initial segment architecture. J Biol Chem. 2017;292(29):12192–207. 10.1074/jbc.M117.784702 PMC5519369 28536263

[39] Braak H, Braak E. Neuropathological stageing of Alzheimer-related changes. Acta Neuropathol. 1991;82(4):239–59. 10.1007/BF00308809 1759558

[40] Zempel H, Mandelkow E. Lost after translation: Missorting of Tau protein and consequences for Alzheimer disease. Trends Neurosci. 2014;37(12):721–32. 10.1016/j.tins.2014.08.004. 25223701

[41] Bachmann S, Bell M, Klimek J, Zempel H. Differential effects of the six human TAU isoforms: Somatic retention of 2N-TAU and increased microtubule number induced by 4R-TAU. Front Neurosci. 2021;15:547. 10.3389/fnins.2021.643115 PMC8185039 34113229

[42] Bachmann S, Linde J, Bell M, Spehr M, Zempel H, Zimmer-Bensch G. DNA methyltransferase 1 (Dnmt1) shapes neuronal activity of human ipsc-derived glutamatergic cortical neurons. Int J Mol Sci. 2021;22(4):1–14. 10.3390/ijms22042034 PMC7922860 33670788

[43] Zempel H, Luedtke J, Mandelkow EM. Tracking Tau in neurons: How to transfect and track exogenous tau into primary neurons. In: Methods in Molecular Biology. New York: Humana Press; 2017. p. 335–40. 10.1007/978-1-4939-6598-4_21 27975261

[44] Fréal A, Rai D, Tas RP, Pan X, Katrukha EA, van de Willige D, et al. Feedback-driven assembly of the axon initial segment. Neuron. 2019;104(2):305–21.e8. 10.1016/j.neuron.2019.07.029 PMC6839619 31474508

[45] Leterrier C. The axon initial segment: An updated viewpoint. J Neurosci. 2018;38(9):2135–45. 10.1523/JNEUROSCI.1922-17.2018 PMC6596274 29378864

[46] Sobotzik JM, Sie JM, Politi C, Del Turco D, Bennett V, Deller T, et al. AnkyrinG is required to maintain axo-dendritic polarity in vivo. Proc Natl Acad Sci U S A. 2009;106(41):17564–9. 10.1073/pnas.0909267106 PMC2765162 19805144

[47] Hedstrom KL, Ogawa Y, Rasband MN. AnkyrinG is required for maintenance of the axon initial segment and neuronal polarity. J Cell Biol. 2008;183(4):635–40. 10.1083/jcb.200806112 PMC2582894 19001126

[48] Kneynsberg A. The role of the axon initial segment and Tau modifications in Axosomatic Tau Distribution. Michigan State University; 2018.

[49] Ichinose S, Ogawa T, Jiang X, Hirokawa N. The spatiotemporal construction of the axon initial segment via KIF3/KAP3/TRIM46 transport under MARK2 signaling. Cell Rep. 2019;28(9):2413–26. 10.1016/j.celrep.2019.07.093 31461655

[50] Nascimento AI, Mar FM, Sousa MM. The intriguing nature of dorsal root ganglion neurons: Linking structure with polarity and function. In: Progress in Neurobiology. Elsevier; 2018. p. 86–103. 10.1016/j.pneurobio.2018.05.002 29729299

[51] Gumy LF, Katrukha EA, Grigoriev I, Jaarsma D, Kapitein LC, Akhmanova A, et al. MAP2 defines a pre-axonal filtering zone to regulate KIF1- versus KIF5-dependent cargo transport in sensory neurons. Neuron. 2017;94(2):347–362.e7. 10.1016/j.neuron.2017.03.046. 28426968

[52] Hamdan H, Lim BC, Torii T, Joshi A, Konning M, Smith C, et al. Mapping axon initial segment structure and function by multiplexed proximity biotinylation. Nat Commun. 2020;11(1):100. 10.1038/s41467-019-13658-5. PMC6941957 31900387

[53] Sun X, Wu Y, Gu M, Liu Z, Ma Y, Li J, et al. Selective filtering defect at the axon initial segment in Alzheimer’s disease mouse models. Proc Natl Acad Sci U S A. 2014;111(39):14271–6. 10.1073/pnas.1411837111 PMC4191768 25232037

[54] Harterink M, Vocking K, Pan X, Jerez EMS, Slenders L, Fréal A, et al. Trim46 organizes microtubule fasciculation in the axon initial segment. J Neurosci. 2019 May;39(25):4864–73. 10.1523/JNEUROSCI.3105-18.2019 PMC6670255 30967428

[55] Yau KW, vanBeuningen SFB, Cunha-Ferreira I, Cloin BMC, vanBattum EY, Will L, et al. Microtubule minus-end binding protein CAMSAP2 controls axon specification and dendrite development. Neuron. 2014;82(5):1058–73. 10.1016/j.neuron.2014.04.019 24908486

[56] Utton MA, Connell J, Asuni AA, van Slegtenhorst M, Hutton M, de Silva R, et al. The slow axonal transport of the microtubule-associated protein tau and the transport rates of different isoforms and mutants in cultured neurons. J Neurosci. 2002;22(15):6394–400, http://www.ncbi.nlm.nih.gov/pubmed/12151518. 10.1523/JNEUROSCI.22-15-06394.2002 PMC6758152 12151518

[57] Kuznetsov IA, Kuznetsov AV. Modeling tau transport in the axon initial segment. Math Biosci. 2020 May;329:108468. 10.1016/j.mbs.2020.108468 PMC8550824 32920097

[58] Farías GG, Guardia CM, Britt DJ, Guo X, Bonifacino JS. Sorting of dendritic and axonal vesicles at the pre-axonal exclusion zone. Cell Rep. 2015 May;13(6):1221–32. 10.1016/j.celrep.2015.09.074 PMC5410646 26527003

[59] Qiang L, Sun X, Austin TO, Muralidharan H, Jean DC, Liu M, et al. Tau does not stabilize axonal microtubules but rather enables them to have long labile domains. Curr Biol. 2018;28(13):2181–9.e4. 10.1016/j.cub.2018.05.045 30008334

[60] Jakobs MAH, Zemel A, Franze K. Unrestrained growth of correctly oriented microtubules instructs axonal microtubule orientation. Elife. 2022;11:e77608. 10.7554/eLife.77608 PMC9550224 36214669

[61] Kollins KM, Bell RL, Butts M, Withers GS. Dendrites differ from axons in patterns of microtubule stability and polymerization during development. Neural Dev. 2009;4(1):1–17. 10.1186/1749-8104-4-26 PMC2717962 19602271

[62] Curcio M, Bradke F. Microtubule organization in the axon: TRIM46 determines the orientation. Neuron. 2015;88(6):1072–4. 10.1016/j.neuron.2015.12.006. 26687215

[63] Ori-McKenney KM, Jan LY, Jan YN. Golgi outposts shape dendrite morphology by functioning as sites of acentrosomal microtubule nucleation in neurons. Neuron. 2012 May;76(5):921–30. 10.1016/j.neuron.2012.10.008 PMC3523279 23217741

[64] Yogev S, Cooper R, Fetter R, Horowitz M, Shen K. Microtubule organization determines axonal transport dynamics. Neuron. 2016 May;92(2):449–60. 10.1016/j.neuron.2016.09.036 PMC5432135 27764672

