# Supplementary material for: Axodendritic targeting of TAU and MAP2 and microtubule polarization in iPSC-derived versus SH-SY5Y-derived human neurons
Source: Open Life Sci. 2024 Dec 31;19(1):20221010. doi: 10.1515/biol-2022-1010 (PMC11699562; doi:10.1515/biol-2022-1010)
Supplement: Supplementary material [file biol-2022-1010-sm.pdf]

Supplementary material

Sequence S1: Sequence of the p_tdtomato-EB3 plasmid.

ttgagatcctttttttctgcgcgtaatctgctgcttgcaaacaaaaaaaccaccgctaccagcggtggtttgtttgccgg

atcaagagctaccaactctttttccgaaggtaactggcttcagcagagcgcagataccaaatactgttcttctagtgtag

ccgtagttaggccaccacttcaagaactctgtagcaccgcctacatacctcgctctgctaatcctgttaccagtggctgc

tgccagtggcgataagtcgtgtcttaccgggttggactcaagacgatagttaccggataaggcgcagcggtcgggctgaa

cggggggttcgtgcacacagcccagcttggagcgaacgacctacaccgaactgagatacctacagcgtgagctatgagaa

agcgccacgcttcccgaagggagaaaggcggacaggtatccggtaagcggcagggtcggaacaggagagcgcacgaggga

gcttccagggggaaacgcctggtatctttatagtcctgtcgggtttcgccacctctgacttgagcgtcgatttttgtgat

gctcgtcaggggggcggagcctatggaaaaacgccagcaacgcggcctttttacggttcctggccttttgctggcctttt

gctcacatgttctttcctgcgttatcccctgattctgtggataaccgtattaccgccatgcattagttattaatagtaat

caattacggggtcattagttcatagcccatatatggagttccgcgttacataacttacggtaaatggcccgcctggctga

ccgcccaacgacccccgcccattgacgtcaataatgacgtatgttcccatagtaacgccaatagggactttccattgacg

tcaatgggtggagtatttacggtaaactgcccacttggcagtacatcaagtgtatcatatgccaagtacgccccctattg

acgtcaatgacggtaaatggcccgcctggcattatgcccagtacatgaccttatgggactttcctacttggcagtacatc

tacgtattagtcatcgctattaccatggtgatgcggttttggcagtacatcaatgggcgtggatagcggtttgactcacg

gggatttccaagtctccaccccattgacgtcaatgggagtttgttttggcaccaaaatcaacgggactttccaaaatgtc

gtaacaactccgccccattgacgcaaatgggcggtaggcgtgtacggtgggaggtctatataagcagagctggtttagtg

aaccgtcagatccgctagcgctaccggactcagatctcgagctcaagcttcgaattctgcagtcgactgccaccatggcc

gtcaatgtgtactccacatctgtgaccagtgaaaatctgagtcgccatgatatgcttgcatgggtcaacgactccctgca

cctcaactataccaagatagaacagctttgttcaggggcagcctactgccagttcatggacatgctcttccccggctgtg

tgcacttgaggaaagtgaagttccaggccaaactagagcatgaatacatccacaacttcaaggtgctgcaagcagctttc

aagaagatgggtgttgacaaaatcattcctgtagagaaattagtgaaaggaaaattccaagataattttgagtttattca

gtggtttaagaaattctttgacgcaaactatgatggaaaggattacaaccctctgctggcgcggcagggccaggacgtag

cgccacctcctaacccaggtgatcagatcttcaacaaatccaagaaactcattggcacagcagttccacagaggacgtcc

cccacaggcccaaaaaacatgcagacctctggccggctgagcaatgtggccccccctgcattctccggaagaatcctcc

atcagcccgaaatggcggccatgagactgatgcccaaattcttgaactcaaccaacagctggtggacttgaagctgacag

tggatgggctggagaaggaacgtgacttctacttcagcaaacttcgtgacatcgagctcatctgccaggagcatgaaagt

gaaaacagccctgttatctcaggcatcattggcatcctctatgccacagaggaaggattcgcacccccctgaggacgatga

gattgaagagcatcaacaagaagaccaggacgagtacctggatccaccggtcgccaccatggtgagcaagggcgaggagg

tcatcaaagagttcatgcgcttcaaggtgcgcatggagggctccatgaacggccacgagttcgagatcgagggcgagggc

gagggccgcccctacgagggcacccagaccgccaagctgaaggtgaccaagggcggccccctgcccttcgcctgggacat

cctgtcccccagttcatgtacggctccaaggcgtacgtgaagcaccccgccgacatccccgattacaagaagctgtcct

tccccgagggcttcaagtgggagcgcgtgatgaacttcgaggacggcggtctggtgaccgtgacccaggactcctccctg

caggacggcacgctgatctacaaggtgaagatgcgcggcaccaacttcccccccgacggccccgtaatgcagaagaagac

catgggctgggaggcctccaccgagcgcctgtaccccgcgacggcgtgctgaagggcgagatccaccaggccctgaagc

tgaaggacggcggccactacctggtggagttcaagaccatctacatggccaagaagcccgtgcaactgcccggctactac

tacgtggacaccaagctggacatcacctcccacaacgaggactacaccatcgtggaacagtacgagcgctccgagggccg

ccaccacctgttcctggggcatggcaccggcagcaccggcagcggcagctccggcaccgcctcctccgaggacaacaaca

tggccgtcatcaaagagttcatgcgcttcaaggtgcgcatggagggctccatgaacggccacgagttcgagatcgagggc

gagggcgagggccgcccctacgagggcacccagaccgccaagctgaaggtgaccaagggcggccccctgcccttcgcctg

ggacatcctgtcccccagttcatgtacggctccaaggcgtacgtgaagcaccccgccgacatccccgattacaagaagc

tgtccttccccgagggcttcaagtgggagcgcgtgatgaacttcgaggacggcggtctggtgaccgtgacccaggactcc

tccctgcaggacggcacgctgatctacaaggtgaagatgcgcggcaccaacttcccccccgacggccccgtaatgcagaa

gaagaccatgggctgggaggcctccaccgagcgcctgtaccccgcgacggcgtgctgaagggcgagatccaccaggccc

tgaagctgaaggacggcggccactacctggtggagttcaagaccatcta-catggccaagaagcccgtgcaactgcccggc

tactactacgtggacaccaagctggacatcacctcccacaacgaggacta-caccatcgtggaacagtacgagcgctccga

gggccgccaccacctgttcctgtacggcatggacgagctgtacaag-taggcggccgcgactctagatcataatcagccat

accacatttgtagaggttttacttgctttaaaaaacctcccacacctccccct-gaacctgaaacataaaatgaatgcaat

tgttgttgttaacttgtttattgcagcttataatggttacaaataaagcaatag-catcacaaatttcacaaataaagcat

ttttttcactgcattctagttgtggtttgtccaaactcatcaatgtatct-taaggcgtaaattgtaagcgttaatatttt

gttaaaattcgcgttaaatttttgttaaatcagctcattttttaaccaataggcc-gaaatcggcaaaatcccttataaat

caaaagaatagaccgagatagggttgagtgttgttccagtttggaacaa-gagtccactattaaagaacgtggactccaac

gtcaaagggcgaaaaaccgtctatcagggcgatggcccactacgtgaac-catcaccctaatcaagttttttggggtcgag

gtgccgtaaagcactaaatcggaaccctaaagggagcccccgattta-gagcttgacggggaaagccggcgaacgtggcga

gaaaggaagggaagaaagcgaaaggagcgggcgctagggcgctgg-caagtgtagcggtcacgctgcgcgtaaccaccaca

cccgccgcgcttaatgcgccgctacagggcgcgtcaggtggcacttttcggg-gaaatgtgcgcggaacccctatttgttt

atttttctaaatacattcaaatatgtatccgctcatgagacaataaccctga-taaatgcttcaataatattgaaaaagga

agagtcctgaggcggaaagaaccagctgtggaatgtgtgtcagt-tagggtgtggaaagtccccaggctccccagcaggca

gaagtatgcaaagcatgcatctcaattagtcagcaaccaggtgtg-gaaagtccccaggctccccagcaggcagaagtatg

caaagcatgcatctcaattagtcagcaaccatagtcccgcccc-taactccgcccatcccgcccctaactccgcccagttc

cgcccattctccgccccatggctgactaattttttttatttatgcagaggcc-gaggccgcctcggcctctgagctattcc

agaagtagtgaggaggcttttttggaggcctaggcttttgcaaagatcgat-caagagacaggatgaggatcgtttcgcat

gattgaacaagatggattgcacgcaggttctccggccgcttgggtgga-gaggctattcggctatgactgggcacaacaga

caatcggctgctctgatgccgccgtgttccggctgtcagcg-caggggcgcccggttctttttgtcaagaccgacctgtcc

ggtgccctgaatgaactgcaagacgaggcagcgcggctatcgtggctggc-cacgacgggcgttccttgcgcagctgtgct

cgacgttgtcactgaagcgggaagggactggctgctattgggc-gaagtgccggggcaggatctcctgtcatctcaccttg

ctcctgccgagaaagtatccatcatggctgatgcaatgcggcggctgca-tacgcttgatccggctacctgcccattcgac

caccaagcgaaacatcgcatcgagcgagcacgtactcggatg-gaagccggtcttgtcgatcaggatgatctggacgaaga

gcatcaggggctcgcgccagccgaactgttcgccaggctcaaggcgag-catgcccgacggcgaggatctcgtcgtgaccc

atggcgatgcctgcttgccgaatatcatggtggaaaatggccgcttttctg-gattcatcgactgtggccggctgggtgtg

gcggaccgctatcaggacatagcgttggctacccgtgatattgctgaa-gagcttggcggcgaatgggctgaccgcttcct

cgtgctttacggtatcgccgctcccgattcgcagcgcatcgccttc-tatcgccttcttgacgagttcttctgagcgggac

tctggggttcgaaatgaccgaccaagcgacgcccaacctgccatcacga-gatttcgattccaccgccgccttctatgaaa

ggttgggcttcggaatcgttttccgggacgccggctggatgatcctc-cagcgcggggatctcatgctggagttcttcgcc

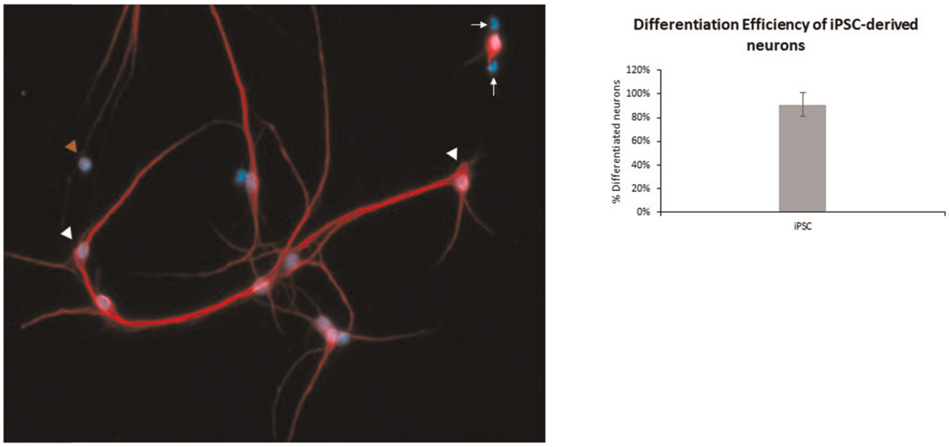

Figure S1: Efficiency of differentiation of iPSC-derived neurons at days 14–18. Assessment of differentiation efficiency of induced pluripotent stem cell (iPSC)-derived neurons at days 14 to 18. Neuronal differentiation was evaluated using the neurons specific marker MAP2 to confirm the presence of mature neurons. Arrows indicate apoptotic cells; white arrow heads indicate differentiated neurons and orange arrow heads indicate undifferentiated cells. The percentage of cells expressing neuronal markers was quantified and presented as [mean ± SEM] from $n = 3$ independent cultures, with 50-100 cells per culture. Images show representative fields from differentiating cultures. Scale bar: 50 µm.

caccctagggggaggctaactgaaacacggaaggagacaataccggaaggaacccgcgctatgacggcaataaaaagaca

gaataaaacgcacggtgttgggtcgtttgttcataaacgcggggttcggtcccagggctggcactctgtcgataccccac

cgagaccccattggggccaatacgcccgcgtttcttccttttccccaccccaccccccaagttcgggtgaaggcccaggg

ctcgcagccaacgtcggggcggcaggccctgccatagcctcaggttactcatatatactttagattgatttaaaacttca

tttttaatttaaaaggatctaggtgaagatcctttttgataatctcatgaccaaaatcccttaacgtgagttttcgttcc

actgagcgtcagaccccgtagaaaagatcaaaggatcttc

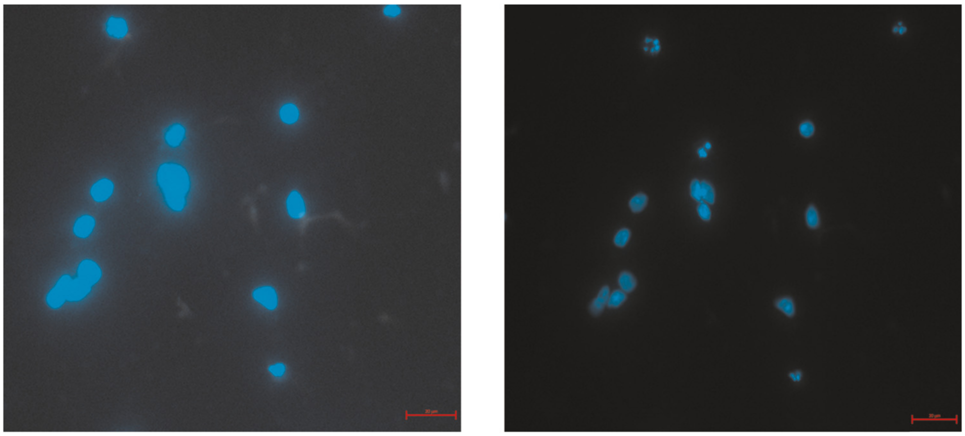

Figure S2: Immunostaining control demonstrating the specificity of secondary antibodies used. Cells were stained with secondary antibodies (anti-mouse, anti-chicken, and anti-rabbit) alone, alongside DAPI as a nuclei marker. The control samples show no significant signal for AnkG, TRIM46, or MAP2, confirming the absence of nonspecific binding. The blue fluorescence from DAPI indicates the presence of nuclei. Scale bars represent 20 µm. exposure times were similar to Figure 2. Left: enhanced contrast (5-fold); right: normal contrast and exposure times used for imaging.

Movie S1: iN d14 EB3-comets dendrites.

Movie S2: iN d14 EB3-comets axon.

Movie S3: SH-SHN d14 EB3-comets dendrites.

Movie S4: SH-SHN d14 EB3-comets axon.

Movie S5: Time lapse videos showing movement of fluorescently tagged EB3 particles in SH-SY5Y-derived neurons (SHN) and iPSC-derived neurons (iN). Neurons were transfected with a plasmid expressing tdTomato-tagged EB3 (ptdTomato-EB3), with SHN cells transfected on day 7 and iN on days 12–15 post-differentiation. EB3-tdTomato expression was observed 2–3 days after transfection. Videos were recorded using a Leica DMi8 S Platform microscope with a Leica DFC9000 camera, capturing images at 1 frame every 2 s over 2 min. Cells were allowed to equilibrate in the microscope life-imaging incubator for 15 minutes before imaging. The frame rate is 10 fps.
